# Supplementary figures and images for: Resurrecting essential amino acid biosynthesis in mammalian cells
Source: eLife. 2022 Sep 27;11:e72847. doi: 10.7554/eLife.72847 (PMC9560156; doi:10.7554/eLife.72847)

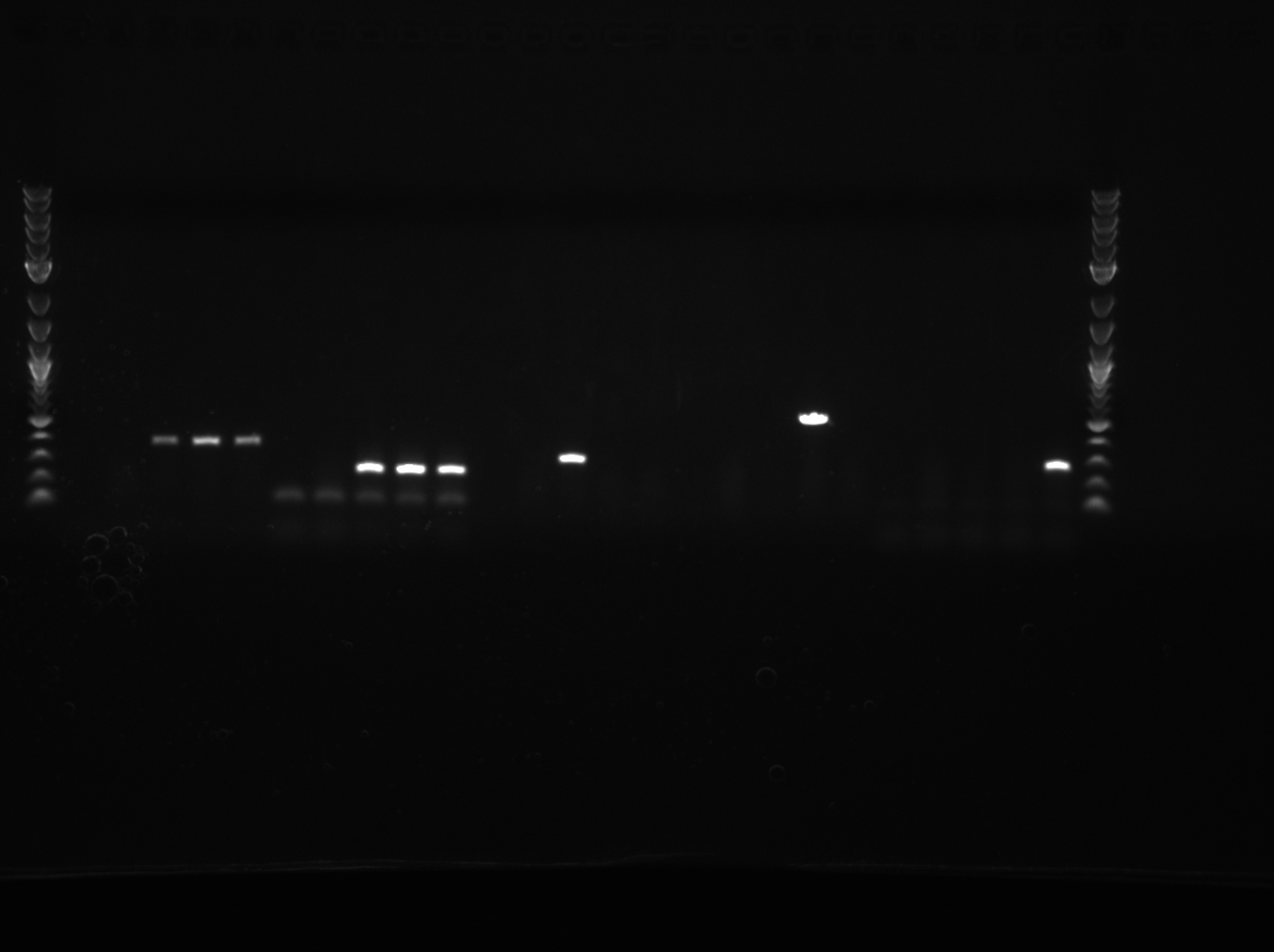

Supplement: Figure 2—figure supplement 2—source data 1. [file elife-72847-fig2-figsupp2-data1.zip › Figure 2-figure supplement 2-source data 1/Figure 2ΓÇôfigure supplement 2-source data 1-raw unedited.tif]

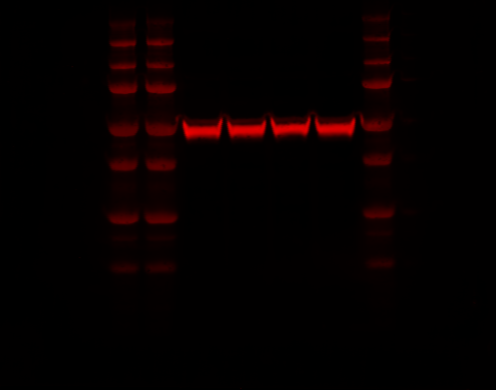

Supplement: Figure 2—figure supplement 2—source data 2. [file elife-72847-fig2-figsupp2-data2.zip › Figure 2-figure supplement 2-source data 2/Figure 2 ΓÇô figure supplement 2C source data 3-raw unedited-Tubulin only.tif]

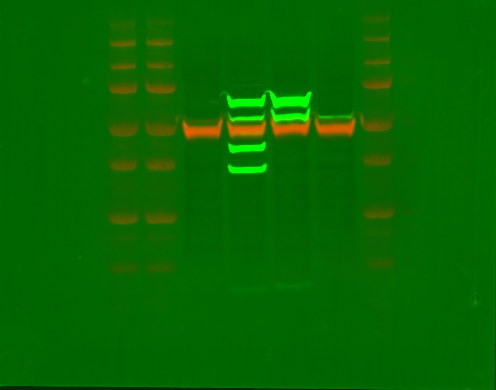

Supplement: Figure 2—figure supplement 2—source data 2. [file elife-72847-fig2-figsupp2-data2.zip › Figure 2-figure supplement 2-source data 2/Figure 2 ΓÇô figure supplement 2C source data 1-raw unedited.tif]

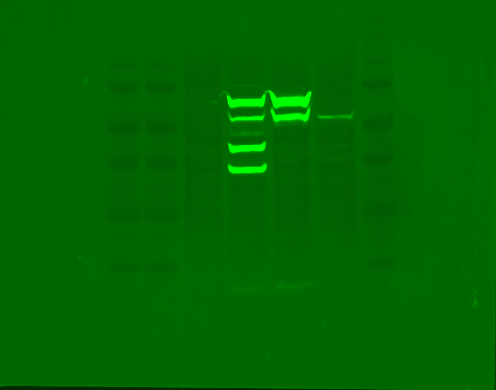

Supplement: Figure 2—figure supplement 2—source data 2. [file elife-72847-fig2-figsupp2-data2.zip › Figure 2-figure supplement 2-source data 2/Figure 2 ΓÇô figure supplement 2C source data 2-raw unedited-P2A only.tif]
